# SOSORT consensus paper: school screening for scoliosis. Where are we today?

**DOI:** 10.1186/1748-7161-2-17

**Published:** 2007-11-26

**Authors:** Theodoros B Grivas, Marian H Wade, Stefano Negrini, Joseph P O'Brien, Toru Maruyama, Martha C Hawes, Manuel Rigo, Hans Rudolf Weiss, Tomasz Kotwicki, Elias S Vasiliadis, Lior Neuhaus Sulam, Tamar Neuhous

**Affiliations:** 1 Orthopaedic Department, "Thriasio" General Hospital, G. Gennimata Av. 19600, Magoula, Attica, Greece; 2 PT Practice, New York, USA; 3 ISICO (Italian Scientific Spine Institute), Milan, Italy; 4 President & CEO, National Scoliosis Foundation (NSF), Boston, USA; 5 Department of Orthopaedic Surgery, Saitama MedicalCenter, Saitama Medical University, 1981 Kamodatsujido, Kawagoe, Saitama 350-8550, Japan; 6 University of Arizona, Tucson AZ 85721, USA; 7 Instituto Èlena Salvá, Barcelona, Spain; 8 Asklepios Katharina Schroth Spinal Deformities Rehabilitation Centre, Bad Sobernheim, Germany; 9 University of Medical Sciences, Poznan, Poland; 10 Bpt physiotherapist specialist in treatment of spinal deformities, Moshe Dayan st. 18 Modiin, 71700, Israel; 11 pt physiotherapist specialist in treatment of spinal deformities, Moshe Dayan st. 18 Modiin, 71700, Israel

## Abstract

This report is the SOSORT Consensus Paper on School Screening for Scoliosis discussed at the 4^th ^International Conference on Conservative Management of Spinal Deformities, presented by SOSORT, on May 2007. The objectives were numerous, 1) the inclusion of the existing information on the issue, 2) the analysis and discussion of the responses by the meeting attendees to the twenty six questions of the questionnaire, 3) the impact of screening on frequency of surgical treatment and of its discontinuation, 4) the reasons why these programs must be continued, 5) the evolving aim of School Screening for Scoliosis and 6) recommendations for improvement of the procedure.

## Background

Early detection of idiopathic scoliosis has been a major and growing commitment of orthopaedists since the early 1960s. A large body of literature, reporting a great deal of clinical experience, has accrued since then [1-364].

G. Dean MacEwen, MD, played an important role in the early development of school screening by implementing programs in all schools in the state of Delaware in the 1960s [43,44,149,162,179,180,237].

The start of screening for scoliosis began in 1963 in Aitken, a town with a population of about 10.000 in central Minnesota [181].

Consequently, the state of Minnesota pioneered spinal screening in the United States by implementing in 1973 a centrally-directed, statewide but voluntary program, based on clinical examination [184].

**Figure 1:**
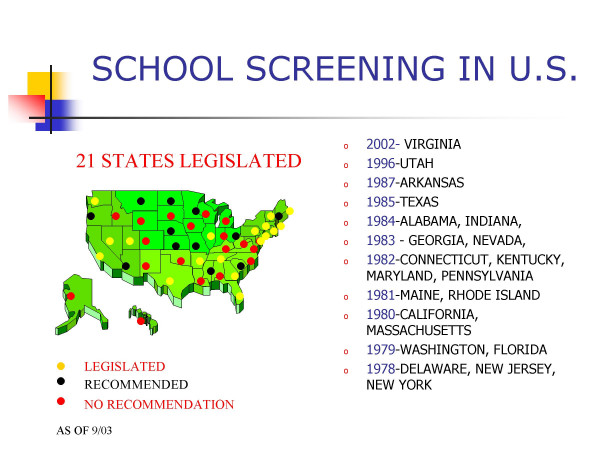
School screening in the USA.

In USA, as of 2003, 21 States had legislated school screening; 11 States recommended school screening without legislation and the remaining either had volunteer screenings or recommended not to conduct screening in the schools, Figure 1.

The 21 legislated States are: 2002-Virginia, 1996-Utah, 1987-Arkansas, 1985-Texas, 1984-Alabama, Indiana, 1983-Georgia, Nevada, 1982-Connecticut, Kentucky, Maryland, Pennsylvania, 1981-Maine, Rhode Island, 1980-California, Massachusetts, 1979-Washington, Florida, 1978-Delaware, New Jersey, New York.

In the USA, not all legislated screening programs are the same today. We can not take a broad-brush approach to whether or not a state has screening, but must look further at screening protocol details, including age and gender screened, screener education and support, scoliometer usage, reporting and follow-up methodologies etc, to evaluate the effectiveness of a program. Some states have ratios of one school nurse for every 700 students, while others have 2000/1 ratios and use health aides and volunteer parents to perform scoliosis screening. Unfortunately, in the USA there is a lack of national standards and adequate reports for scoliosis screening mechanisms, making collection of evidence-based outcomes necessary to either enhance this process or eliminate it, extremely difficult. Perhaps a study should be first done to compare the legislative states and document the intra and inter state variability of screening programs and outcome results [332,333].

Japan is the nation with a federally mandated screening program, mostly accomplished with surface topography using the moiré technique and low-dose roentgenographic techniques [275].

In Japan, school-screening program for scoliosis is mandatory by law. But actual program depends on local educational committees, as they take charge of their ownprogram. The local educational committee is set up at each city and ward. Some committees use moiré topography, while others don't. Therefore, the rate of the committees using moiré topography in Japan is unclear. With respect to the Tokyo area, the committees which use moiré surface topography are less than half [334].

The British Orthopaedic Association and the British Scoliosis Society conclude that it should not be a national policy to routinely screen children for scoliosis throughout the United Kingdom [50].

The fact that school screening programs for scoliosis remain the subject of considerable controversy is a not a new issue [58,79,80,114,154,181,182,203,207,286,296,299,304,323,326,329,330].

Over-referral of adolescents with insignificant curves led to a marked decrease in many programs, because screening was reported to have a low positive predictive value (0.05 percent). It was demonstrated that over-referral is common, even when multiple diagnostic modalities are used [164,326].

That conclusion was based, in part, on the lack of studies documenting improved outcomes from early identification and treatment of children with scoliosis. While subsequent reports have both supported and questioned the effectiveness of brace treatment for scoliosis [9,210,220], by that time, no randomized, prospective studies have clarified the efficacy of brace treatment.

The American Academy of Orthopedic Surgeons recommends screening girls at ages 11 and 13, and screening boys once at 13 or 14 years of age.

The American Academy of Paediatrics has recommended scoliosis screening with the Adam's forward bending test at routine health visits at 10, 12, 14 and 16 years of age, although evidence does not exist to support these recommendations.

In 1996, the U.S. Preventive Services Task Force released its opinion on screening for adolescent idiopathic scoliosis. The Task Force noted that 'there is insufficient evidence for or against routine screening of asymptomatic adolescents for idiopathic scoliosis. [291,330].

Subsequently, in 2004, the US Preventive Service Task Force recommended against the routine screening of asymptomatic adolescents for idiopathic scoliosis. US Preventive Service Task Force changed its recommendation and advised against screening, not on the grounds of new evidence, but by changing the methodology of rating the existing evidence [292,293]!

School screening was introduced sporadically in Australia and to a variable extent in most states and territories. By the early 1990s the cost factor led to the abandonment of most programs in government schools and a new strategy was sought after. The Spine Society of Australia, with the support of the Royal Australian College of General Practitioners, introduced The National Self-Detection Program for Scoliosis. In essence, this entails the distribution of a simple brochure to the target age group (11–13 years of age) in which the outward signs of scoliosis are described. If, after reading the brochure, a girl or her parents think she may have a curvature, then follow-up with the family doctor is recommended. An educational program on scoliosis for general practitioners was also introduced via *The Australian Family Physician *and is available on the website [335].

This policy resulted in a shortage of recent publications on prevalence studies and therefore in a lack of any research on aetiology in these south latitudes, in contrast to the results and outcome studies that originated from school screening programs from countries located at north latitudes [130].

Studies on aetiology could ultimately help in designing a reasonable aetiological treatment. Recent prevalence studies based on school screening programs, published at least in English language, are also lacking from Africa and South America.

Spurred by the ongoing controversy about this universal and fundamental issue, SOSORT included this consensus paper on school screening in the 4^th ^Society's Meeting in order to study, analyze, understand and finally recommend on school screening.

The following colleagues namely Joseph P. O'Brien, Martha C. Hawes, Toru Maruyama, Stefano Negrini, Manuel Rigo, Elias S. Vasiliadis, Marian H. Wade, Tomasz Kotwicki and Hans Rudolf Weiss contributed in some way to the improvement of the initial questionnaire, which was created by the chairman of this consensus paper. For the full Consensus Paper see Additional file 1.

Before the discussion of this paper at the Boston SOSORT meeting, the questionnaire was completed by 10 colleagues, namely Lorenzo Aulisa, Martha C. Hawes, Toru Maruyama, Stefano Negrini, Manuel Rigo, Elias S. Vasiliadis, Hans Rudolf Weiss, Lior Neuhaus Sulam and Tamar Neuhous, Tomasz Kotwicki and the chairman.

The preliminary results gave an initial impression about the current understanding on the issue. However, after the adjourn of the 4^th ^Boston SOSORT Meeting, 3–7 May 2007, and the end of the relevant consensus paper on the school screening, thirty five completed forms were collected from colleagues representing thirteen countries, namely Austria (one), Canada (one), France (one), Germany (one), Greece (one), Israel (five), Italy (three), Japan (one), Norway (three), Poland (five), Spain (one), UK (four), USA (eight). The results of these completed forms are presented and discussed in this report.

The professions of the attendees were orthopaedic surgeons (six), orthopaedic surgeons and physiatrists (two), rehabilitation doctors (five), physiotherapists and orthopaedic surgeons (two), physiotherapists (nine), nurse (one), C.P.O. (one), various other health care providers (nine).

The Consensus Paper on school screening consisted of 26 questions, see Additional file 1.

Each question (Q) is deemed with Consensus (100 – 51% positive answers), with no Consensus (49 – 0% positive answers respectively).

For definitions related to screening see Additional file 2.

## The primary questions

Q1 Title: proposed titles of the consensus paper

The first question (**Q1**) was on the proposed titles of the consensus paper which were:

a) *School Screening for Scoliosis. Where are we today? Proposal for a consensus,*

b) *School Screening *and

c) *School Screening for posture in Children*.

*Results*: For the **Q1 **there were 32 positive answers for the question a (91.4%), 2 for b (5.7%) and one for c (2.8%). This point (Question 1 for the title) has Consensus.

*Discussion: *A point that could be raised with the title is the conformation with a 'consensus.' In fact, in this paper there is no unanimous consensus, but we found, as it will be shown in the next pages, that there are different habits, and experiences, and we do not close with statements that define which consensus we have. Or, better, there is consensus on some answers. Therefore this study is combining the philosophy of consensus and the description of what the widely accepted title is referring to: *"School Screening for Scoliosis. Where are we today? Proposal for a consensus." *Therefore, this paper is rather a proposal for, not a solid consensus, and also describes the present status on the issue.

*Q2 Title: Do you perform a school-screening program for scoliosis at your center*?

*Results*: For the **Q2 **there were 9 yes (25.7%), 25 no (71.4%) and 1 (2.8%) don't know. Yes (Italy, Greece, Japan, USA), No (Austria, Canada, France, Germany, Israel, Norway, Poland, Spain, UK).

*Discussion: *This question has no consensus.

*Q3 Title: *(*Is any form of screening for scoliosis performed at your place of work (e.g., organizations, like orphanages, boarding schools, universities, hospitals, factories, offices, etc.*)?

*Results*: For the **Q3 **there were 12 yes (34.3%), 21 no (60%) and 2 don't know (5.7%). A responder notes also that (the) paediatricians check the childrens' back at 6 (years), 10 (years) and 14 years of age.

*Discussion: *Looking at Q2 and Q3, it appears that more centres perform school screening at the above countries than was initially believed.

Q4 Title: Has a previously run school-screening program been discontinued at your place of work?

*Results*: For the **Q4 **there were 7 yes (20%), 24 no (68.6%) and 4 don't know (31.4%).

*Discussion*: It is interesting to quote the various comments accompanying the answer to this question like: 'Parents are usually angry when they discover that their child could be diagnosed earlier, before visiting the health provider. Parents are happy with school screening. School screening was a routine and stopped because of money. Parents demand again and again to have it.' 'Screening was interrupted because of the absence of public health administrators' interest.' 'Both parents and school physicians were sorrowful for the interruption of the program that was felt as a good opportunity for the health of the children.' 'In speaking with school nurses in the area who used to participate in screening, their impression was that little follow-up was done in response to their work – even if parents took their children to physicians, the physicians did not recommend follow-up or treatment.' 'We use to see surgical cases at first visit never discovered before, coming only from south Italy. Now we see them also from the north. People with scoliosis are angry at this.' 'Not to my knowledge but I cannot answer this 100% sure.' '15 years ago there was a school screening as a routine at the ages of 6, 10, 12–13, performed by school nurses; since then, because of economic problems, most of the school nurses tasks cancelled, including the posture screening. Today there is no school screening as a routine but here and there it is performed by school doctors or nurses but it is not a law. As a result, there are many cases of late and misdiagnosed and many parents are angry about it. The financial problems in the education system obliged for other priorities.'

Q5 Title: a = Is your center or practice actually screening per the request of a school or public health department, or b = are you doing it on your own as a gesture of good will?

*Results*: For the **Q5***)*, there were 3 readings for a (8.6%), 10 readings for b (28.6%), 4 for both a and b (11.4%) and 28 don't know (51.4%).

*Discussion: *The following comment was reported: 'We tried a few times to perform the screening voluntarily but every time there was objection from some sores.'

This question has no consensus.

## Organization

There is great diversity in the policies for scoliosis screening worldwide. For example, in the United States there is no nationwide requirement or standard for scoliosis screening; such policies are established at the local state, county, city, or school district level. In some states, scoliosis screening may be done in the pediatrician's office or by a chiropractor or other health care professional, and not in the schools at all. Because scoliosis screening policies are so variable in different parts of the world, we are asking the following questions.

Q6 Title: Is the school screening program compulsory in your country, state or local area?)

*Results: *For the **Q6 **(there were 8 yes (22,85%) 26 no (74.28%) and 1 don't know (2.85%). Yes (Japan), No Italy, USA, Italy, Spain, Greece, Germany, Israel and Poland

*Discussion: *Therefore there is a vague picture on the issue of school screening in Japan, Europe, North America, but there is no information from Africa, Australia and Latin America.

This question has no consensus.

Q7 Title: Which authorities are responsible for providing permission for school screening in your country?

*Results: *For the **Q7**, there were the following answers for the various countries:

**Austria, **Ministry. **Canada, **Health Canada. **France**, Ministry of Health. **Germany**, Ministry. **Greece**, Greek Ministry of Education, the Hospital Board of Directors applies to the Greek Ministry of Education for permission to enter the schools and perform the program. **Israel **Ministry of Education, Ministry of Health and the Head of the School. **Italy**, local referent. In **Italy **would be the same as in Greece. **Japan**, Local Education Committee. **Norway**, Health Directory of the Ministry of Healthcare. **Poland**, Ministry and local authorities (also Ministry of Education for permission to enter the schools and perform the program). Ministry of Education, Ministry of health, local department of education in the municipality and the head of the school. **Spain**, Ministry. **UK**, Ministry. **USA**, Ministry, State mandated, Board of education, the Public Health Agencies of the Federal and State.

*Discussion: *This question has no consensus.

Q8 Title: Who pays for the screening program?

*Results: *For the **Q8 **there were the following answers for the various countries:

**Austria, Canada, France**, **Germany**, **Greece**, Indirectly the Hospital, at which the School screening program team is working. **Israel, **Nobody. **Italy, **Nobody. **Japan, **Local educational committee. **Norway**, **Poland, **Nobody. **Spain**, **UK**, **USA, **State, Govermment.

*Discussion: *For the discussion on cost for school screening please see also **Q23 **[20,124,129,133,206].

This question has no consensus.

Q9 Title: Is the (Orthopaedic) Medical Association of your country or state supporting the school screening program?

*Results: *For the **Q9 **there were the following answers for the various countries: Yes = 5 (14.28%), no = 15 42.85%, there is no statement on the issue = 5 (14.28%), I don't know 10 (28.57%).

*Discussion: *Only 14% of *Orthopaedic or Medical Associations of the participants countries are supporting school screening.*

Our recommendation would be the communication with National Orthopaedic or Medical Associations in order to explain the problem to them and convince them to issue a positive statement for school screening examination.

This question has no consensus.

*Q10 Title: Would you support a change of the term **school screening ***to ***postural screening****?*

*Results: *For the **Q10**, there were No = 20 (57.15%) and yes = 15 (42.85%).

*Discussion: *In, many places in the United States the nomenclature for school screening has been changed to **Postural Screening**, because the exam involves looking at postural deviations to identify the presence of scoliotic, kyphotic, and/or lordotic deformities.

The comments accompanying the answer of this question were very important and indicative for no change, 'as posture can be impaired without deformity, therefore there is a need to search for deformity, and not for bad posture that counts for less than 50% during school age,' also 'posture pertains to physical education and not health services.' Moreover, 'school screening is a general term and could include many topics and some other screening examinations e.g. epidemiological data, anthropometric measurements, which are extremely necessary in scoliosis research.' 'The term postural screening is restricted to postural measurements and thus is inadequate.' It was also stated that 'postural screening is a specialized activity that should be kept to specialist,' or 'school screening is a more appropriate description and everyone knows what it means.'

However, the arguments for a change are also reasonable. They state that it takes the emphasis away from 'school' and may help change the misconception that if the school doesn't do it there is no need for it to be done at all. Also, it helps detection of Scheuermann in boys aged 12–14 years.

This question has no consensus.

## Methods and criteria used for school screening: what happens afterwards for children at risk

*Q11 Title: *a. *who performs school screening currently at your center? and b. who do you think would be the most appropriate persons to perform the screening?*

*Results: *For the **Q11 **a and b, the response from the attendees is described in Table 1.

**Table 1:** The school screening performers

**Health care provider**	**Q11a**	**Q11a (%)**	**Q11b**	**Q11b (%)**
Orthopaedic doctors (**OD**)	6	**17,14**	8	**22,85**
Health Care Visitors (**HCV**)	1	02,85	6	**17,14**
Registered Nurses (**RN**)	2	05.71	10	**28,57**
Physical therapists (**PT**)	10	**28,57**	27	**77,14**
Physical Education Teachers (**PET**)	4	**11,42**		
School Nurses (**SN**)	17	**48,57**	18	**51,42**
Other			2	05.71
Combination of				
I don't know	13	37,14		
Spine Specialist (SS)			1	02,85
School Doctor (SD)	1	02,85		

Q11a represents the number of answers on the questionnaires for question a. *Who performs school screening currently at your center? *and Q11a (%) the relevant percentages.

Q11b represents the the number of answers on the questionnaires for question *b*. *Who do you think would be the most appropriate persons to perform the screening? *and Q11b (%) the relevant percentages.

*Discussion: *As it appears from the answers of this questionnaire, the school screening performers internationally are currently: **SN**s 48.57%, **PT**s 28,57%, **OD**s 17.14%, **PET**s 11,42%, and follow in percentage of involvement **RN**s, **SD**s, and **HCV**s. However, the most appropriate persons to perform the screening were considered to be in descending preference the **PT**s 77.14%, **SN**s 51.42%, **RN**s 28.57%, **OD**s 22.85%, **HCV**s 17.14%, and follow in percentage of involvement the SS. **PET**s were not considered at all as school screening performers.

There is variety in the personnel performing the school screening. The main issue in performing the program is training of the personnel. There are some training programs, like the one of the Arkansas Department of Health, which provide an Instructor Training Course in Scoliosis Screening. This workshop is designed to teach the principles and proper technique for scoliosis screening. Any graduate of this course is a Certified Scoliosis Screening Instructor and is qualified to teach persons to be scoliosis screeners. The certification is valid for a period of five years, after which time an update course in Scoliosis Screening is required for recertification. Recertification is again for a five year period and this cycle continues.

This question has no consensus.

### Definitions concerning personnel training

A. **Certified Instructors: **Individuals who train the screeners. These shall be licensed health practitioners who have successfully completed (for example the Arkansas Department of Health Instructor) a Training Course in Scoliosis Screening.

B. **Screeners: **Individuals who perform the actual scoliosis screening. These shall be licensed physicians, individuals who have been trained to perform scoliosis screening by a Certified Scoliosis Screening Instructor or individuals who can document completion of a Scoliosis Screening Workshop within the past five years and demonstrate competence to a Certified Scoliosis Screening Instructor level [11,336].

*Q12 a and b Title: *a) *What is the sex and age range of those screened at your center? b)Please indicate below the respective sexes and age ranges that you think should be screened.*

*Results: *For the **Q12a **and **Q12b **answers see Table 2.

**Table 2:** Sex and age range of those screened currently

**Age girls**	**Q12a**	**Q12a (%)**	**Q12b**	**Q12b (%)**
Younger than 7 years of age	4–14 years = 1 5–10 years = 1		4–14 years = 1 5–15 years = 1 5, 6 years = 2	
7 years	6	17,14	7	20,00
8 years	4	11,42	6	17,14
9 years	5	14,28	13	**37,14**
**10 years**	8	**22,85**	19	**54,28**
**11 years**	7	**20,00**	20	**57,14**
**12 years**	11	**31,42**	19	**54,28**
**13 years**	7	**20,00**	17	**48,57**
**14 years**	7	**20,00**	7	**20,00**
15 years	2	05,71	4	11,42
Older than 15 years	2	05,71	1	02,85
All school ages			1	02,85
No answer	17	48,57%		

**Age boys**	**Q12a**	**Q12a (%)**	**Q12b**	**Q12b (%)**

Younger than 7 years of age	4–14years = 1		4–14 years = 1 5–17 years = 1 5, 6 years = 2	
7 years	6	17,14	6	17,14
8 years	5	14,28	5	14,28
9 years	6	17,14	5	14,28
**10 years**	9	**25,71**	10	**28,57**
**11 years**	7	**20,00**	12	**34,28**
**12 years**	10	**28,57**	17	**48,57**
13 years	6	17,14	19	**54,28**
**14 years**	7	**20,00**	15	**42,85**
15 years	2	05,71	8	**22,85**
Older than 15 years	2	05,71	2	05,71
All school ages			1	02,85
No answer	17	48,57%		

Q12a represents the number of answers on the questionnaire for question a. *What is the sex and age range of those screened at your center? *and Q12α (%) the relevant percentages, while Q12b represents the number of answers on the questionnaire for question *b Please indicate below the respective sexes and age ranges that you think should be screened, *and Q12b (%) the relevant percentages.

*Discussion: *The development and mainly the progression of adolescent idiopathic scoliosis (AIS) are related in girls to the appearance of menses. This biological milestone does not always appear at similar ages in girls living in different geographical latitudes. Therefore, the age ranges that should be screened must be in accordance with this variation. However, if it is feasible to screen wider age ranges, then the collected information could give valuable aetiological clues, more reliable longitudinal data and a consequent improvement of our insight to the pathophysiology of AIS. Considering that there are no sufficient epidemiological data in the literature for the prevalence of idiopathic scoliosis in several geographical areas and the natural history is not yet accurately predictable, we can assume that school screening is not only an instrument for early detection and decrease in the number of adolescents that will eventually experience operative treatment, but is also a priceless tool for research on scoliosis aetiology [121-123,125-127,130,172,173,262,263,270,271].

This question has no consensus.

Q13 Title: Is a scoliometer used during the screening examination at your center?

*Results: *For the **Q13 **there were the following answers: Yes n = 23 (65.71%), no n = 3 (08,57%), I don't know n = 9 (25,71%).

*Discussion: *The *Bunnell scoliometer *is widely used (by 19 users). The *Prujis scoliometer *(by 2 users) is also used. The recommendation is the use of a scoliometer for the performance of school screening.

Question 13 has consensus.

Q14 Title: While performing the forward bending test (FBT), in what position is the scoliometer measurement taken*?

*Results: *For the **Q14 **Standing FBT n = 17 (48,57%), Sitting FBT n = 4 (11,42%), Prone non, both Standing FBT and Sitting FBT n = 6 (17,14%).

Discussion: * The standing forward bending test (FBT) traditionally refers to the Adams Forward Bending Test; however, some additional body positions have been utilized recently, i.e., the sitting or prone positions. For this reason, we are herein substituting the terms Standing FBT, Sitting FBT or Prone Position for the Adams Forward Bending Test.

All screening techniques depend on surface topography. The Adams forward bending test is well known to school and primary health care personnel and widely used to provide a subjective or qualitative evaluation of spinal deformity. The application of physical measurements provides a quantitative evaluation of deformity and the basis for objective referral criteria for screening, which substantially increases its effectiveness [10].

In the standing forward bending position, the student is asked to bend forward looking down, keeping the feet approximately 15 cm apart, knees braced back, shoulders loose and hands positioned in front of knees or shins with elbows straight and palms opposed.

Many devices and techniques have been used, including measurement of the rib hump height using a level and ruler, stereophotogrammetry, flexicurve, ultrasound, thermography, back contour devices, etc. Moiré topography, a photographic method, and computerized surface mapping systems such as the Integrated Shape Imaging Systems (ISIS) have been studied extensively and provide the most complete description of surface topography. The time and expense required to do these studies make them impractical for mass screening. Inclinometry [measurement of the angle of trunk rotation (ATR) observed with the patient in the forward bent position] seems to be the simplest, quickest, most reliable, and least expensive objective measurement of trunk deformity. One useful device, the scoliometer (Orthopaedic Systems, Inc., Union City, CA), has achieved widespread usage with numerous reports of its reliability. None of these techniques is diagnostic. Radiographs are required to establish the diagnosis, aetiology, and severity of spinal deformity [4,6,43,45,74,135,164,172,190,247,289].

This question has no consensus, even thought it is close to it.

Q15 Title: Are any signs of maturity documented while screening?

*Results: *For the **Q15 **There were Yes n = 7 (20.00%), No n = 15 (42,85%) and no information n = 13 (37%).

*Discussion: *It was reported that the menarche state, only if the patient is scoliosis positive, is the only documented sign of maturity.

This question has no consensus.

Q16 Title: Has your center encountered non-cooperation or refusal of the screening examination from children or their parents?

*Results: *For the **Q16 **there were Yes n = 3 (8,57%), No n = 15 (42,85%), no information n = 17 (48,57%).

*Discussion: *The responders reported that 2% refused school screening. This should be considered an acceptable rate, as many children are present at the following year's screening. It was also reported that the encountered difficulties in performing the school screening were not usually the non-cooperation or refusal of the screening examination by parents but mainly the negative attitude from mainly older children. The parental refusal was included in the consent form filled earlier by them. The examiners can usually do nothing about it.

Q17 Title: Over which ATI (Angle of Trunk Inclination) or ATR (Angle of Trunk Rotation) is a hospital consultation and/or radiographical examination recommended?

*Results: *For the **Q17 **the response was:

More than 4 degrees of ATI/ATR n = 1 (2,85%), more than 5 degrees of ATI/ATR n = 10 (28,57%).

More than 6 degrees of ATI/ATR n = 8 (22,85%), more than 7 degrees of ATI/ATR n = 2 (05,71%).

More than 8 degrees of ATI/ATR n = 1 (2,85%), other: Scoliometer exam by itself is not enough to decide, no information n = 13 (37,14%).

*Discussion: *For the discussion of this question see also questions 13, 18 and 20.

This question has no consensus.

Q18 Title: Where are those who need to be referred sent for further assessment?

*Results: *For the **Q18 **the response was:

To our hospital n = 7 (20%), to any specialized outpatient department of any hospital in our city/state/country n = 6 (17,14%), to our private practice n = 4 (11,42%), to our hospital or to any specialized outpatient department of any hospital in our city/state/country n = 1 (2,85%), to any specialized outpatient department of any hospital in our city/state/country or to our private practice n = 2 (5,71%), to no one; the family is sent a note which encourages them to schedule a visit with their pediatrician n = 1 (2,85%), to the primary physician n = 1 (2,85%), to an orthopaedic doctor n = 1 (2,85%), to family doctor n = 1 (2,85%).

*Discussion: *The AAOS and the SRS maintain that not all children who are referred because of a positive screening result require radiography [7]. By design, school screening will refer some children who do not have scoliosis in an effort not to miss referring children with scoliosis. The question of when to obtain radiography cannot be answered on the basis of available scientific data. It is recommended that it is likely to obtain radiographs in children who have (1) a large, unambiguous curve on physical examination, (2) asymmetry on examination in skeletally immature children (the risk of curve progression is greatest during growth), (3) asymmetry on examination and a family history of scoliosis, and (4) asymmetry and neurologic signs or symptoms [9]. The scoliometer threshold reading of 7 degrees or more is used by the majority of practitioners, [122]. It appears, however, that age and growth play an important role in the agreement of scoliosis surface and radiological deformity [131,132].

This question has no consensus.

Q19 Title: Please describe the treatment offered to those referred from the screening program:

*Results: *For the **Q19 **the response was:

Observation, with timing and frequency thereof: (three times a year, or three months during growth spurt), Exercises, and when prescribed: (if possible defect exist), Brace treatment, and when prescribed: (25° Cobb angle, progressed curves, and depend on the case and doctor, above 25° Cobb and immature, According to Italian guidelines [214,311,312].

*Discussion: *This question has no consensus.

Q20 Title: Please fill in this table with data from your screening experience (if available):

*Results: *For the **Q20 **the response was:

a. Number of people screened: .............. in the year(s) ..................................

b. Percentage ofscoliosis detected in the screened sample: ...........

ATR threshold for scoliosis detection: ............

c. Percentage of patients radiographed: .............

ATR threshold for radiographic prescription: .............

d. Percentage of patients with prescription of exercises: ..........

e. Percentage of patients with prescription of a brace: .............

g. Percentage of patients with prescription of surgery: .............

In this question we are quoting the teams' response screened n = 1000 or more students.

Number of people screened: 15000 in the year(s) 1983–1994.

Number of people screened: 10000......... in the year(s) 1997–2006.

Number of people screened: 9995 in the year(s).

Percentage ofscoliosis detected in the screened sample 7%.

Percentage ofscoliosis detected in the screened sample: 2.9%.

Percentage ofscoliosis detected in the screened sample: 2%.

ATR threshold for scoliosis detection: 4.

ATR threshold for scoliosis detection: ≥7 degrees.

ATR threshold for scoliosis detection: 5°.

Percentage of patients radiographed: 80%.

Percentage of patients radiographed: 3.5%.

Percentage of patients radiographed: .2%.

ATR threshold for radiographic prescription: 5°.

ATR threshold for radiographic prescription: ≥7 degrees.

ATR threshold for radiographic prescription: 5°.

Percentage of patients with prescription of exercises: 80%.

Percentage of patients with prescription of exercises:

Percentage of patients with prescription of exercises:

Percentage of patients with prescription of a brace: 10%.

Percentage of patients with prescription of a brace: 0.3%.

Percentage of patients with prescription of a brace: 1%.

Percentage of patients with prescription of surgery: 0%.

Percentage of patients with prescription of surgery: 0.03%.

Percentage of patients with prescription of surgery:

*Discussion: *The responses pertained to children screened and not to known patients (i.e. scoliosis already screened).

Q21 Title: Do you believe that school screening is useful for clinical purposes, i.e., does it affect the age at which scoliosis is treated?

*Results: *For the **Q21 **there were Yes n = 31 (88,57%), No n = 0 (0%), Not sufficient data n = 1 (2,85%), not sure n = 1 (2,85%)

*Discussion: *This question has consensus.

Q22 Title: Do you believe that school screening is a valuable undertaking, even though the aetiology of idiopathic scoliosis is not yet clear and an aetiologically-based treatment has not yet been established?

*Results: *For the **Q22: **Yes n = 31 (91,42%), No n = 1 (2,85%).

*Discussion: *The reasons given were numerous: 'to determine prevalence in the population, and to direct patients to appropriate care'; 'it is not a big price to find and save one boy from school and save from surgery'; 'an early effective treatment will minimize progression and therefore reduce the number of surgeries and number of people with severe curves'; 'it would ensure more favorable outcomes and reduce deformity, minimize surgery and would make economic sense'; 'epidemiology data, early treatment effectiveness'; 'early detection improves final results'; 'enables an early diagnosis of the disease and an early treatment'; 'besides its clinical usefulness, it is a valuable research tool.'

A more eloquent report is adduced because it beautifully analyses the issue: "If screening is eliminated, we will return to the old days when the 'usual time of diagnosis' [203] is forty degrees or more, an obvious deformity is present, and surgery can be justified without further ado. Otherwise, citizens suffering from mild or moderate spinal deformity will continue to remain in the dark about the possible basis for their pain, pulmonary limitations and psychological problems. The current absence of any meaningful therapies for spinal deformity through the medical community does not make screening 'unethical,' as Dickson and Weinstein [80] have proposed. Instead, this situation makes it all the more important that patients be in a position to help themselves. The only legitimate reasons not to screen for a condition with the potential to cause a lifelong struggle with a range of health problems are (1) To avoid overexposure to X-rays, and (2) To avoid overtreating children with traumatic interventions of dubious clinical value, like bracing and spinal fusion surgery. These dangers are restricted to the context of current approaches as applied in this country. Given the limitations of the Cobb angle for judging spinal deformity in a meaningful way, ongoing work by some researchers to establish more appropriate and less destructive clinical assays perhaps is the most critical area of needed research [115,331,337-344]."

It was also stated by another responder that current knowledge about scoliosis and conservative management thereof is not enough among the general body of specialists to take advantage of early detection. Lack of knowledge is related to misleading information reaching the patients and their families, abuse of non-effective therapies, abuse of invasive tests (X-ray) and finally over-treatment. However, this situation could be alleviated by education provided by specialists from, for example, the SOSORT and ultimately the situation could be reverted: right information, effective light therapies, X-ray only when necessary and prevention of under-treatment.

The negativists are stating that 'cost too much,' 'most of the cases that we treat from below 20 Cobb under 12 years, more than 99% do not come to surgery.'

The current knowledge is that early recognition of the disease and appropriate conservative treatment, when indicated, changes the natural history of idiopathic scoliosis. There is adequate evidence in the literature to support this statement [345-356]. Torell et al [285], evaluated the effect of a program for early detection and treatment of idiopathic scoliosis in a stable population of 1.5 million over a ten-year period. Seven hundred and twenty-five patients with a scoliosis of more than 20 degrees (as measured with Cobb's method) and aged twenty years or younger were followed during that period. Although treatment principles remained essentially the same, the percentage of patients who required an operation decreased each year. The magnitude of the ten most severe curves detected each year decreased from an average of 64 degrees to 44 degrees. Efforts to detect scoliosis early have resulted in a threefold increase in the number of patients treated for scoliosis [285].

Korovesis et al. [171], in a prospective study of the effect of TLSO on spinal deformities, indicated that TLSO treatment halted the progression of scoliosis and reduced the number of patients requiring surgery, thus changing the natural history of the disease.

Nachemson et al. [210] described the results of a prospective multi-centre analysis of 286 girls with AIS and documented that bracing altered the natural history by preventing curve progression.

Rowe et al. [245], in a meta-analysis of 37 peer-reviewed articles on conservative treatment of scoliosis, found that 23-hour bracing was the most successful means of halting progression of scoliosis; the Milwaukee brace was found to have the highest rate of success in comparison to electrical stimulation.

The beneficial results of the brace on the natural history of the above curves were also described by Valavanis et al. [294]. The results and the reliability of the brace were subsequently checked and confirmed by Grivas et al. [128].

Therefore it is evident that bracing prevents about 20% to 40% of appropriately braced curves from progressing 6° or more [17].

This point (Question 22) has Consensus

Q23 Title: Do you believe that the concept of cost-benefit analysis should be applied to screening?

*Results: *For the **Q23 **there were Yes, n = 17 (48.57%), No n = 15 (42,85%), Don't know n = 2 (5,71%).

*Discussion: *The reasons given for **yes**:

a) Unfortunately in our country we have limited resources for both education and health care. Therefore, benefits of screening must be weighed against costs.

b) For evaluation purposes.

c) As a medical doctor I would like to say NO, but this would be too idealist and I think the world cannot go on without the application of cost-benefit analysis, when public funds are used. However, any program supported exclusively by NGO could be done without the application of this concept.

d) Undetected cases >30° are rare, however physicians should know more!

e) Yes but not exclusively and not decisively.

The reasons given for **no**:

a) Because scoliosis can have profound effects on individual's life.

b) First I think about the individual, the public system doesn't take into consideration the one boy or girl who needs the treatment.

c) Again one saving, one avoid the surgery, will equal a screening.

d) In the end, the reduced number of surgeries and scoliosis back pain-related treatments will save the government more money than screening would cost.

e) If 1 or 2 local children per year have the option to avoid surgery, this is a justification for the program.

f) I think cost-benefit analysis is meaningless given the absence of tracking, standards, and follow-up of patients. Thus, for example, a single patient who receives multiple revision surgeries costing hundreds of thousands of dollars skews all analyses and none of these are considered in 'cost-benefit' analysis.

g) Because the cost of a late treatment surpasses the cost of an early treatment.

h) You have to try to do it at low cost.

j) You cannot apply cost-benefit principles to research that originate from school screening.

k) The one case that can be saved from surgery has a 100% success rate for that particular case.

l) Early detection implies early treatment and by that less surgery. Thus, increased costs at an early stage may decrease later costs [133,184,206,285].

As a common thinking, in order to achieve reasonable cost effectiveness, the number of false-positive referrals must be reduced and minimize the cost and radiation of the smaller curves [67].

For definitions related to direct and indirect school screening cost, the reader is referred to the definition section.

A realistic evaluation of both direct and indirect costs is not feasible and could result in inaccurate overestimation of the total cost as it might take into consideration many qualitative and subjective factors, such as the definition of scoliosis, the threshold for referrals for radiological evaluation, indications for conservative and operative treatment, cost to the society, children's compliance, decisions of the clinicians, effectiveness of treatment and impact on children's quality of life [20,133,179,285].

The negativists of school screening implicate the increased indirect cost and the psychological impact on the child, which basically cannot be measured, to criticise these programs. However, they are not discussing about the cost of the child's and his/her family psychological stress when a severe, untreated curve is discovered, or the cost of the child's and family's psychological stress when they are led to the operating room, or even what the feeling of an operated scoliotic is, with a rod in her back holding him/her straight. No one has given a frank answer on this issue so far. As Dr. Bunnell characteristically reports, 'we're not looking for the cheapest way to screen – we're looking for a better quality outcome for our patients'. Additionally, we must be motivated and guided by an ancient Greek saying, according to which 'it is better to prevent than to treat'.

This point (Question 23) has no Consensus.

Q24 Title: Do you believe that school screening is useful for academic purposes, i.e., do we learn about scoliosis from school screening?

*Results: *For the **Q 24 **there were Yes n = 26 (74,28%), No n = 2 (5,71%), I don't know 7 (20%).

*Discussion: *The reasons given for No were:

a) The big problem is adolescent scoliosis, b) not the prevalence, but progression rate in the long-term is important, c) previous research has shown it not to be beneficial, so why continue.

In cases of yes, it was further asked to describe the knowledge that has been acquired thus far or that can be advanced from the performance of school screening:

The reasons given for yes were:

a) Prevalence of AIS school children.

b) Increase in the understanding of scoliosis.

c) Statistical analysis.

d) Teach MDs.

e) It will provide a more accurate understanding about the age scoliosis appears at – rather than taking onset as the time of detection or first diagnosed data.

d) It would be more helpful if accompanied by a demographics history form.

e) Prevalence of scoliosis.

f) students learn about scoliosis.

g) Most of what we have learned about incidence is based on school screening.

h) Prevalence, early detection, prevention of progression.

i) Research on natural history and aetiopathogenesis could be designed much better.

j) Knowledge of scoliosis incidence, influence of genetic or environmental factors in scoliosis aetiology.

k) Perform longitudinal studies.

l) How form and function change with the development of scoliosis.

m) We can study epidemiology of scoliosis.

n) The results of school screening programs provide valuable data regarding the prevalence and the natural history of idiopathic scoliosis.

o) The benefits of scoliosis screening include increased public awareness of and knowledge about epidemiology and natural history of scoliosis.

p) Considering that there are no sufficient epidemiological data in the literature for the prevalence of idiopathic scoliosis in several geographical areas and the natural history is not yet accurately predictable, we can assume that school screening not only is a means of early detection and diminution of the number of adolescents that will eventually experience operative treatment, but it is a priceless tool for research on scoliosis aetiology as well [9,45,121-123,125-127,172,173,179,184,237,262,263,270,271].

Question 24 has Consensus.

Q25 Title: What do you think of exploring the prevalence of scoliosis throughout the lifespan?

*Results: *For the **Q 25 **Yes, it would be useful to explore the prevalence of scoliosis throughout lifespan. n = 20 (57,14%), No, it would not be useful to explore the prevalence of scoliosis throughout lifespan. n = 7 (20%), I don't know 7 (20%).

*Discussion: *It was thought that by asking only about the utility of screening adolescents, we might miss an opportunity to expand the scope of our communal thinking about scoliosis screening. Although numerous studies suggest that the prevalence of scoliosis is much higher in the elderly population than among adolescents, epidemiological studies to date have focused almost exclusively on paediatric populations. Given the potential toll of a progressive asymmetrical deformity on the ability of elderly people to maintain their balance and avoid falls, it would appear that an expansion of our focus to include some exploration of the prevalence of scoliosis throughout lifespan would be justified [231,254].

The comments for **Yes **were:

• Because it is a chronic illness.

• Scoliosis progresses through life especially for women. People with scoliosis tend to have many back problems and pain as they age.

• Because functional scoliosis can become structural and greatly impact quality of life.

• I have no knowledge about this but see adults with scoliosis secondary to osteoporosis. I believe adults should do similar screening every year.

• At our clinic we are contacted by many elderly people whose scoliosis has either never been detected or is late-onset.

• I think there are many adults suffering from scoliosis with no one to help them.

• Scoliosis at any age is functional and reversible in its early stages, its appearance has aetiological implications for children and grandchildren and it makes no sense to avoid diagnosis and education.

• This is one of the most effective tools to check when prevention policies are effective.

• It would be useful to investigate to what percentage adult scoliosis is different from idiopathic scoliosis of adolescents and is not caused by degenerative spinal disorders or segmental spinal instability.

• If a high prevalence of scoliosis throughout lifespan will be found, the health community should research and learn more about scoliosis, in order to treat functional problems and back problems.

The comments for **No **were:

• No need to treat these patients unless they have symptoms, in which case they will visit a doctor.

• The big problem is adolescent scoliosis.

• Not prevalence, but progression rate in the long-term is important.

• Screening of adolescents is useful to prevent the deformity. In the elderly, treatment is reserved only for symptomatic curves that do not need a screening program.

• The problem is how (there are no problem places where you find the entire population like schools) and consequently who and where.

• We know about scoliosis 'de novo' in adulthood, so what more?

• It is not useful to explore the prevalence of scoliosis throughout the lifespan. Pain is the primary sign and will bring the patient to the doctor; deformity will be diagnosed on this occasion.

This Question 25 has Consensus.

Q26 Title: Please add any issue relevant to the survey or any question that you consider to be important and think should be added to the questionnaire.

*Results: *For the **Q 26 **there were the following suggestions and/or comments:

• I am glad that the SOSORT is looking at the issues of screening in both the childhood and adults

• a) Screening b) Spine specialist consulting for moderate to severe scoliosis. I work outside the government and medical systems. I only know about screening through my students. This survey was difficult for me to respond to. Some Yoga teachers are highly trained in postural analysis and may be helpful resource for performing the screening.

• It is very important to decide how to do the screening. One by one in the 'hung room' or in the middle of the class? Take shorts off or not? Ask the child about pain or other subject?

• I believe that school screening should be compulsory in all countries – as we can only come to fully understand the complicities of scoliosis

• I would like to see school screening in the UK

• It seems to me better to improve the school nurse.

• In families or individuals with known risk factors (there are hundreds – including childhood surgery or trauma, birth injuries, familial disorders with a high prevalence of scoliosis, infections, spinal injuries, etc), screening for scoliosis should occur routinely and often throughout life, and research to develop simple, nondestructive methods to reverse curvatures before they become deformities, needs to be a high priority.

• In the 5 years after beginning screening (1988–1999) there has been only 1 surgical treatment in this area for scoliosis.

• At least 3 years in girls and boys differently have to be evaluated, because no one really knows when the growth spurt will start. And when it starts, 12 months' time can be too late (I have seen 5 – 50° in 3 Months!). Which means, in order to be on the safe side, 11–13 (maybe 10 years) year old girls and 12–14 year old boys should be screened every 3 months; does anybody find that reasonable?

## The impact of scoliosis school screening on frequency of surgical treatment

The earlier data reported on this issue appear to be in some way inconsistent and inconclusive. For example, in Minnesota, USA, a place with school screening in practice, a decreasing frequency of IS surgery was found, beginning in 1974 and continuing through 1979, the last year reported, [184]. Torell et al. [285] reported that scoliosis school screening, (SSS), reduced the number of surgically treated IS patients.

In a different report [11], data on the frequency of surgical treatment per thousand children screened for 7 or more years, were disclosed from three US states: Kansas and Virginia showed no clear trend. For Minnesota, the frequency of surgery was decreasing until 1981–82, after which it increased [11].

Some more recent European reports are more convincing on the impact of conservative treatment on the frequency of surgical treatment of IS. The incidence/prevalence of surgery can significantly be reduced where high-standard conservative treatment is available [189,239,314,345-356].

## The impact of discontinuation of scoliosis school screening

The consequences of discontinuation of school scoliosis screening programs on the referral patterns of AIS patients remain unknown. A recent cross-sectional study was conducted of all patients referred for suspected adolescent idiopathic scoliosis (AIS) for an initial visit to the orthopaedic outpatient clinic of a metropolitan paediatric hospital in Canada [25]. The objective was to document the appropriateness of current referral patterns for AIS in comparison to those that prevailed before discontinuation of school screening in Canada. Of the 489 referred cases, suspected of having AIS, 206 (42%) had no significant deformity (Cobb angle <10 degrees) and could be considered as inappropriate referrals. In subjects with confirmed AIS, 91 patients (32%) were classified as late referrals with regards to brace treatment indications. The authors conclude that current referral mechanisms for AIS are leading to a suboptimal case-mix in orthopedics in terms of appropriateness of referral [25].

The fact is that this was widely expected and the triumph of epidemiology over early diagnosis was in reality a disaster. Prevention must be a standard policy in civilized societies with medical systems caring about people's well-being and not about statistics, epidemiology or only money. We always have to remember what the axiom in the cradle of western civilization, ancient Greece, was. Ancient Greeks used to say that 'metron of everything is man'; the measure, in other words, of appraising everything is only the human being, nothing else.

## Why we must continue school screening programs

It is reported [145] that the policy not to screen because of lack of cost effectiveness is based on the obsolete assumption, derived from an early study [255], that surgery is the only proven treatment option. As pointed out by Hawes [145], the cited study does not justify scientifically this conclusion. Today there is evidence that signs and symptoms of scoliosis can indeed be changed after the application of an intensive in-patient exercise programme [311], and that the rate of progression can be reduced significantly [313]. Furthermore, the incidence/prevalence of surgery can significantly be diminished where conservative treatment is available at a high standard [189,239,314]. It has also been documented and is generally accepted that bracing does alter the natural history of idiopathic scoliosis [17,128,171,210,245,285,294,357] and school screening does reduce the number of surgically treated IS patients, as discussed above [285].

Studies on psychosocial health and body image have revealed that functioning in these domains may affect compliance behaviour and satisfaction with treatment outcomes among adolescent patients. Psychosocial and body image disturbance is less marked in patients with good social or family functioning, or patients who exercise regularly or are psychologically healthy [284].

Taken together, these studies support the hypothesis that school screening is justified to allow to detect mild and reversible spinal curvatures and treat them conservatively before they develop into spinal deformities with a potential to cause symptoms throughout life [146].

By no means should we aim at replacing school screening by costly methods of gene screening; these are probably useful in predicting curve progression but concordance is far less than 90% in monozygotic twins and phenotypic variability seems to be very high [166,310].

## The evolving aim of scoliosis screening

The goal of scoliosis screening is to detect scoliosis at an early stage, when the deformity is likely to go unnoticed and there is an opportunity for a less invasive method of treatment, or less surgery, than would otherwise be the case [70,184,206,285].

What in reality scoliosis school screening program does, using the scoliometer or any other surface measuring device, is reveal children with surface, mainly thoracic surface, deformity. It does not reveal the scoliosis per se. It is now definitely accepted that the surface deformity does not accurately predict the magnitude of scoliosis, especially in younger children. As Bunnell characteristically states [45], 'it has become apparent from many reports that, although there is a significant correlation between clinical deformity and radiographic measurement, the standard deviation is so high that it is not possible to reliably predict the degree of curvature from surface topography in any given patient by any technique'. It has also been reported that, in typical screening settings where the prevalence and positive predictive value are relatively low, for every curve >10° detected, there are 1–5 false-positives; similarly, for every curve >20° detected, there are 3–24 false-positives [131,132,134,291].

The above described phenomenon of over-referrals from school screening programs is the cause of the burden and of the ongoing controversy over its application. Therefore, it must be widely accepted that, with school-screening programs, a chance is mainly given to the school-aged population to rule out those who will be at risk for developing scoliosis, rather than discover those who definitely have scoliosis. This is especially true if there is a significant surface deformity justifying the central axis (that is the spinal) deformity. There is something else that must be highlighted and clearly understood. The school screening program aims at detection of surface deformity and/or the existing number of scoliosis cases; it does not aim at predicting which scoliotic curves will progress to a type that will require some type of conservative or surgical treatment. The criteria used to predict progression of a small or moderate curve are unfortunately not related to school screening programs. All asymmetric children, therefore, who will be entitled to develop scoliosis will miss the opportunity to be picked up and will probably be discovered too late, when surgery will be the only treatment option. As expected, the outcome will be particularly worse in poor societies. Therefore, in explaining the role of school screening, it must also be clearly understood that its cost must be the direct cost of performance of the actual screening program and not the subsequent expenditures of follow-up, radiographs and other modalities described in the current literature [44,133].

## Recommendations for improvement of school screening procedure

In addition to our recommendations stated in the discussion of the pertinent questions, Dr Bunnell's recommendations [44,45] for improvement of the school screening procedure are also quoted here.

Recommendations for improvement include redefinition of what actually constitutes a 'significant' scoliosis for screening, diagnostic, and outcome purposes; selective screening of only immature females; the use of objective referral criteria; and re-screening patients rather than referring borderline cases [358].

Dr Bunnell claims that spinal screening programs must have defined referral criteria and "treatment-eligible" degrees of scoliosis, in order to judge their effectiveness. The ideal criteria will minimize both the number of referrals and the number of false-negative examinations. In view of the new prevalence data from his study and the current recommendations to wait until scoliosis approaches 30 degrees (Cobb angle) before starting brace treatment, he recommends changing the screening referral criterion to seven degrees ATR at any level of the spine and changing the definition of false-negative (treatment-eligible curves that are missed) to 30 degrees Cobb angle for the purposes of spinal screening. Under these recommendations, it is anticipated to accomplish a referral rate of 3% and detect 95% of all "treatment eligible" curvatures, thus preserving an acceptably low false-negative rate and helping maintain cost effectiveness of spinal screening programs. For youngsters whose curves are below the "treatment eligible" line – for example between 20 and 25°, a repeat screening is recommended within six to twelve months. Repeat screening would take place at school, thus keeping it a public health issue. In conclusion, Dr Bunnell states that *'screening is vitally important, but we do not want to screen out a whole bunch of people who don't need medical attention because it's very costly. We're not looking for the cheapest way to screen – we're looking for a better quality outcome for our patients.'*

Grivas et al. [359], by reviewing the collective experience of the "Thriasio" school screening program, provide specific evidence-based recommendations for the improvement of school screening effectiveness.

School screening has to be set up on a district basis and held by a team of experienced examiners who will organize and prepare everything well in advance. All the interested parties must be informed by distribution of informative material and lectures. Prior to the visit of the examining group to the school, the parents must fill out a consent form and the pupils must fill out a particular form regarding their personal and demographic data.

The regression curve of both the IS prevalence and age at menarche by geographical latitude is following a parallel declining course, especially in latitudes northern than 25 degrees; this means that in northern latitudes, girls experience late age at menarche and higher prevalence of IS. IS almost always occurs during the time of peak growth velocity, typically during the year just prior to menarche. Therefore, in order to increase the predictive value of school screening, we should screen girls who live in northern countries at an older age range than those who live in the south.

By screening children in the sitting position with the use of a scoliometer, we can dramatically decrease the number of referrals, because we eliminate the effects of leg length inequality and pelvic obliquity on the spine. Sitting position reveals the true trunk asymmetry which could be associated with IS and therefore is recommended as a standard examination method in a school screening program.

The referral process must be standardized according to a specific protocol by documenting all the prognostic factors for progression of a detected curve. As a second stage of screening, demographic and clinical parameters, including the gender, the chronological age, the age at menarche, the pattern and the magnitude of asymmetry and the growth potential must be recorded, in order for the more experienced Orthopaedic surgeons to determine whether it is necessary to x-ray a referred child or not.

Approximately 25% of younger referred girls (aged <13 years old) with an ATR ≥ 7° were found to have either a straight spine or a spinal curve under 10°. In this age group the correlation between clinical deformity and radiographic measurement is not statistically significant, while in older referred girls (aged 14–18 years old) it is. Therefore, all the younger individuals who are identified with a surface deformity but without a severe scoliotic curve are at risk for IS development and need to be kept under observation and not discharged from regular follow-up.

It is crucial for everyone who participates to fully recognize the voluntary basis of the program, in order to reduce the financial cost. The financial cost can be either direct or indirect. There is no general consensus among economists as to what constitutes the indirect cost in a cost-effectiveness analysis, because the indirect cost cannot be measured precisely, as it is related to the effectiveness of the school screening program. A more effective screening program has lower indirect cost. Therefore, the economic information on screening for scoliosis which is available to decision-makers should mainly be based on studies of the direct cost of such programs. The direct cost of a screening program can be reduced to a minimum, if it is well organized and performed on a voluntary basis, according to the model of the "Thriasio" school screening program.

## Authors' contributions

TBG created the initial questionnaire, chaired this consensus paper at the Boston SOSORT Meeting, May 13–15, 2007, processed the collected data, collected the literature and contributed in drafting of the manuscript. The following colleagues namely JPO'B*, MCH*, TM*, SN*, MR, ESV*, MHW*, TK* and HRW*, LNS, TN contributed in some way to the improvement of the initial questionnaire. HRW and ESV partially contributed in drafting of the manuscript. *These authors contributed by reviewing, text editing and adding certain text files and references. All authors have read and approved the final manuscript.

## Supplementary Material

Additional file 1 Consensus paper on school screening questionnaire. The data provided include the complete consensus paper on school screening questionnaire. Click here for file

Additional file 2 Term definitions pertinent to school screening. The data provided represent definitions for terms related to school screening. Click here for file
