# Supplementary material for: SOSORT consensus paper: school screening for scoliosis. Where are we today?
Source: Scoliosis. 2007 Nov 26;2:17. doi: 10.1186/1748-7161-2-17 (PMC2228277; doi:10.1186/1748-7161-2-17)
Supplement: Additional file 1 — Consensus paper on school screening questionnaire. The data provided include the complete consensus paper on school screening questionnaire. [file 1748-7161-2-17-S1.doc]

**Additional file 2**

**Consensus paper on school screening questionnaire.**

**Consensus paper on school screening**

Chairman: TB Grivas (Greece)

Please read the following questions carefully and choose the answer you consider to be the most suitable for you and your country or state. If you have any questions, suggestions or information that you would like to add, please do so at the end of the questionnaire. Those of you who may not be able to answer the questions pertaining to clinicians, but who have an interest in scoliosis research or management, are specifically invited to skip down to answer only those questions that may apply to you or for which you have an interest or opinion.

# **1) Proposed titles of the consensus paper. Which do you prefer?**

(Please check)

- School Screening for Scoliosis. Where are we today? Proposal for a consensus.
- School Screening.

#### **THE PRIMARY QUESTIONS**

# **2) Do you perform a school-screening program for scoliosis at your center?**

# Yes

# No

Please write your city and country or state and details about the center.

…………………………………………………………………………………………………

…………………………………………………………………………………………………

# **3) Is any form of screening for scoliosis performed at your place of work (*e.g.*, organizations**

# **like orphanages, boarding schools, universities, hospitals, factories, offices, etc.)?**

# Yes

# No

# Don’t know

If yes, please describe:

……………………………………………………………………………………………………

……………………………………………………………………………………………………

# **4) Has a previously run school-screening program been discontinued at your place of work?**

- Yes
- No

If yes, please describe why it was discontinued. Also, in your opinion, what were the results of discontinuing the program, in clinical and scientific terms? Did you receive any feedback from parents, schools, health professionals, or others regarding the discontinuation of school screening? If yes, what was their reaction?

……………………………………………………………………………………………………

……………………………………………………………………………………………………

……………………………………………………………………………………………………

……………………………………………………………………………………………………

# **5) Is your center or practice actually screening per the request of a school or public**

# **health department, or are you doing it on your own as a gesture of good will?**

- Our center performs screening per the request of a school or public health department.
- Our center performs screening voluntarily as a gesture of good will.

#### **ORGANIZATION**

There is great diversity in the policies for scoliosis screening worldwide. For example, in the United States there is no countrywide requirement or standard for scoliosis screening; such policies are established at the local state, county, city, or school district level. In some states, scoliosis screening may be done in the pediatrician’s office or by a chiropractor or other health care professional, and not in the schools at all. Because scoliosis screening policies are so variant in different parts of the world, we are asking the following questions.

**6) Is the school screening program compulsory in your country, state or local area?**

- Yes
- No

**7) Which authorities are responsible for providing permission for school screening in**

**your country?** (For example, in Greece, the Hospital Board of Directors applies to the Greek

Ministry of Education for permission to enter the schools and perform the program.)

………………………………………………………………………………………………………………………………………………………………………………………………………………

**8) Who pays for the screening program?**

………………………………………………………………………………………………………………………………………………………………………………………………………………

**9) Is the (Orthopaedic) Medical Association of your country or state supporting the school**

**screening program?**

- Yes
- No
- There is no statement on the issue

Comments:……………………………………………………………………………………….…………………………………………………………………………………………………………

In many places in the United States the nomenclature for school screening has been changed to **Postural Screening**, because the exam involves looking at postural deviations to identify the presence of scoliotic, kyphotic, and/or lordotic deformities.

**10) Would you support a change of the term *school screening* to *postural screening?***

- Yes
- No

Comments: ………………………………………………………………………………………………………

………………………………………………………………………………………………………

………………………………………………………………………………………………………

**METHODS & CRITERIA USED FOR SCHOOL SCREENING: WHAT HAPPENS AFTERWARDS FOR CHILDREN AT RISK?**

**11a) Who performs school screening currently at your center?**

- 1.Orthopaedic doctors
- 2. Health care visitors
- 3. Registered nurses
- 4. Physical therapists
- 5. Physical education teachers
- 7. School nurses
- 8. Other (please write): ……………………………………………………………..
- 9. Combination of: ........…………………………… (for example, 1 and 2, or other)

**11b) Who do you think would be the most appropriate persons to perform the screening?**

- 1.Orthopaedic doctors
- 2. Health care visitors
- 3. Registered nurses
- 4. Physical therapists
- 5. Physical education teachers
- 7. School nurses
- 8. Other (please write): ……………………………………………………………..
- 9. Combination of: ........…………………………… (for example, 1 and 2, or other)

**12a) What is the sex and age range of those screened at your center?**

(Please check all that apply. If both sexes are screened at the same age range, please

skip down to the combined "Both Girls and Boys" section.)

##### **Girls**

- Younger than 7 years of age (please indicate from what age on): …………
- 7 years
- 8 years
- 9 years
- 10 years
- 11 years
- 12 years
- 13 years
- 14 years
- 15 years
- Older than 15 years (please indicate up to what age): ...............

##### **Boys**

- Younger than 7 years of age (please indicate from what age on): …………
- 7 years
- 8 years
- 9 years
- 10 years
- 11 years
- 12 years
- 13 years
- 14 years
- 15 years
- Older than 15 years (please indicate up to what age): ..............

##### **Both Girls and Boys**

- Younger than 7 years of age (please indicate from what age on): …………
- 7 years
- 8 years
- 9 years
- 10 years
- 11 years
- 12 years
- 13 years
- 14 years
- 15 years
- Older than 15 years (please indicate up to what age): ..............

**12b)z Please indicate below the respective sexes and age ranges that you think**

**should be screened.**

(Please check all that apply. If you think that both sexes should be screened at

the same age range, please skip down to the combined "Both Girls and Boys" section.)

Girls

- Younger than 7 years of age (please indicate from what age on): …………
- 7 years
- 8 years
- 9 years
- 10 years
- 11 years
- 12 years
- 13 years
- 14 years
- 15 years
- Older than 15 years (please indicate up to what age): ...............

##### **Boys**

- Younger than 7 years of age (please indicate from what age on): …………
- 7 years
- 8 years
- 9 years
- 10 years
- 11 years
- 12 years
- 13 years
- 14 years
- 15 years
- Older than 15 years (please indicate up to what age): ..............

##### **Both Girls and Boys**

- Younger than 7 years of age (please indicate from what age on): …………
- 7 years
- 8 years
- 9 years
- 10 years
- 11 years
- 12 years
- 13 years
- 14 years
- 15 years
- Older than 15 years (please indicate up to what age): ..............

**13) Is a scoliometer used during the screening examination at your center?**

- Yes
- No

If yes, please indicate the type of scoliometer used (*e.g.*, Prujis, Bunnell, etc.).

- Prujis
- Bunnell
- Other (please describe): .….………..………………..…………………………….

**14) While performing the forward bending test (FBT), in what position is the scoliometer**

**measurement taken*?**

- Standing FBT
- Sitting FBT
- Prone
- Combination of (please list): ……………………………………………………………

*The standing forward bending test (FBT) traditionally refers to the Adams Forward Bending Test; however, recently some additional body positions have been utilized; i.e., the sitting or prone positions. For this reason, we are herein substituting the terms Standing FBT, Sitting FBT or Prone Position for the Adams Forward Bending Test.

**15) Are any signs of maturity documented while screening?**

- Yes
- No

If yes, please describe what signs of maturity you are documenting (*e.g*., Tanner stage or other).

………………………………………………………………………………………………………

………………………………………………………………………………………………………

**16) Has your center encountered non-cooperation or refusal of the screening examination**

**from children or their parents?**

- Yes
- No

How is the problem handled?

……………………………………………………………………………………………..………..

…………………………………………………………………………………………………........

……………………………………………………………………………………………….……...

**17) Over which ATI (Angle of Trunk Inclination) or ATR (Angle of Trunk Rotation) is a**

**hospital consultation and/or radiographical examination recommended?**

- More than 5 degrees of ATI/ATR
- More than 6 degrees of ATI/ATR
- More than 7 degrees of ATI/ATR
- More than 8 degrees of ATI/ATR
- Other: .....…………………………………………………………………………………..

**18) Where are those who need to be referred sent for further assessment?**

- To our hospital
- To any specialized outpatient department of any hospital in our city/state/country
- To our private practice
- Other (please indicate) ………………………………………………………………..

**19) Please describe the treatment offered to those referred from the screening program:**

- Observation, with timing and frequency thereof: ………………………………………..
- Exercises, and when prescribed: …………………………………………………………
- Brace treatment, and when prescribed: ……………………………………….………….
- Other (please describe): ………………………………………………………………….
- Don’t know

**20) Please fill in this table with data from your screening experience (if available):**

Number of people screened: .............. in the year(s) ..................................

Percentage of scoliosis detected in the screened sample: ...........

ATR threshold for scoliosis detection: ............

Percentage of patients radiographed: .............

ATR threshold for radiographic prescription: .............

Percentage of patients with prescription of exercises: ..........

Percentage of patients with prescription of a brace: .............

Percentage of patients with prescription of surgery: .............

**21) Do you believe that school screening is useful for clinical purposes; *i.e.*, does it affect**

**the age at which scoliosis is treated?**

- Yes
- No

**22) Do you believe that school screening is a valuable undertaking, even though the**

**aetiology of idiopathic scoliosis is not yet clear and an aetiologically-based treatment**

**has not yet been established?**

- Yes
- No

Please give a reason:

……………………………………………………………………………………………………

...………………………………………………………………………………………………….

**23) Do you believe that the concept of cost-benefit analysis should be applied to screening?**

- Yes
- No

Please give a reason:

……………………………………………………………………………………………..………

………………………………………………………………………………………………………

….………………………………………………………………………………………………….

……………………………………………………………………………………………………….

**24) Do you believe that school screening is useful for academic purposes; *i.e.*, do we learn**

**about scoliosis from school screening?**

- Yes
- No

If yes, please describe the knowledge that has been acquired thus far or that can be advanced from the performance of school screening: ………………………………………………………………………………………………………………………………………………………………………………………………………………………………………………………………………………………………………………………

…………………………………………………………………………………………………….

To ask only about the utility of screening adolescents may be to miss an opportunity to expand the scope of our communal thinking about scoliosis screening. Although numerous studies suggest that the prevalence of scoliosis is much higher in the elderly population than among adolescents, epidemiological studies to date have focused almost exclusively on the prevalence of scoliosis in children. Given the potential toll of a progressive asymmetrical deformity on the ability of elderly people to maintain their balance and avoid falls, it would appear that an expansion of our focus to include some exploration of the prevalence of scoliosis throughout the lifespan would be in order.

**25) What do you think of exploring the prevalence of scoliosis throughout the lifespan?**

- Yes, it is useful to explore the prevalence of scoliosis throughout the lifespan.
- No, it is not useful to explore the prevalence of scoliosis throughout the lifespan.

Please comment:

………………………………………………………………………………………………………

……………………………………………………………………………………………………..

………………………………………………………………………………………………………

**26) Please add any issue relevant to the survey or any question that you consider to be**

**important and think should be added to the questionnaire.**

………………………………………………………………………………………………………………………………………………………………………………………………………………………………………………………………………………………………………………………………………………………………………………………………………………………………

………………………………………………………………………………………………………

………………………………………………………………………………………………………

###### **ADDENDUM**

### The issue of school screening for scoliosis is not settled universally or in the United States.

### SRS/AAOS Position Statement, School Screening Programs for the Early Detection of Scoliosis: The AAOS and the Scoliosis Research Society continue to support the principle of school screening for scoliosis[362]. *https://www.srs.org/members/statements/school_screening.asp*

The U.S. Preventive Services Task Force recently has provided an update[292, 293] of its 1993 conclusions that were based on *Woolf SH 1993 JAMA 269: 2664-2666* [363]*.*

Screening for AIS, policy statement and *Woolf SH 1993 JAMA 269: 2667-2672.*  Screening for AIS, review article:

In the 1993 study[330], the committee concluded that sufficient information was not available, based on the published record, to recommend for or against screening.

In 2004, an updated survey available at <http://www.ahrq.gov/clinic/3rduspstf/scoliosis/scolioup.htm> [364] concluded that there still is no compelling evidence for or against the premise that early screening is beneficial, and recommended that early detection be considered not justified.

The answers to the questionnaire will be reported as pooled data only, *i.e*., the individual respondent's answers will be kept confidential.

**Questionnaire** **respondent’s demographics**

Name: ……………………………………………………………………………………………

Surname: …………………………………………………………………………………………

Specialty: …………………………………………………………………………………………

I am working under the:

- National (public) health system
- Private sector

Institution’s name: ……………………………………….……………………………………...

Address: …………………………………………………………………………………………

Country/State: ………………………………………………………………………………….

e-mail: …………………………………………………………………………………………..

Fax: ……………………………… Tel: ………………………………………………..

The original document on school screening has been edited to date from input offered by:

1. Hans Rudolf Weiss
2. Toru Maruyama
3. Stefano Negrini
4. Elias S. Vasiliadis
5. Martha C. Hawes
6. Joseph P. O'Brien
7. Manuel Rigo
8. Marian H. Wade

Please return your completed forms to:

Dr. Theodoros B. GRIVAS, MD

Orthopaedic & Spinal Surgeon

D. Bernardou 31 str, Brilisssia, 15235, Athens, Greece, E-mail: [grivastb@vodafone.net.gr](mailto:grivastb@vodafone.net.gr) or [grivastb@hol.gr](mailto:grivastb@hol.gr)
