# Supplementary material for: SOSORT consensus paper: school screening for scoliosis. Where are we today?
Source: Scoliosis. 2007 Nov 26;2:17. doi: 10.1186/1748-7161-2-17 (PMC2228277; doi:10.1186/1748-7161-2-17)
Supplement: Additional file 2 — Term definitions pertinent to school screening. The data provided represent definitions for terms related to school screening. [file 1748-7161-2-17-S2.doc]

**Additional file 1**

**Definitions for terms related to school screening. A word.doc file.**

**Screening:** A systematic examination or assessment, done especially to detect an unwanted substance or attribute. (<http://www.answers.com/Screening>)

# **Screening:** Examination of people with no symptoms, to detect unsuspected disease.

<http://cancerweb.ncl.ac.uk/cgi-bin/omd?query=screening>

**Screening:** Organized periodic procedures performed on large groups of people for the purpose of detecting disease. (<http://www.online-medical-dictionary.org/omd.asp?q=screening>)

**Scoliosis Screening Procedure:** The procedure used, usually at school, to examine a child for scoliosis. It consists of evaluating the child in various positions, with the forward bend technique being the principal one.

**Terms for the assessment of screening tests**

The validity of the test can be measured by three parameters: the **sensitivity,** or the ability of the test to select those with the disease; the **specificity,** or the ability of the test to correctly identify those who do not have the disease; and the **predictive value,** that is the frequency with which a positive test actually signifies the disease (**Table 1**) [360].

However, knowing the sensitivity and specificity of the test is not enough. What is important is what the test yields. This can be expressed as the **positive predictive value,** or the frequency with which a positive test actually identifies the disease (**Table 1**) [203, 360].

**Definitions**

**Sensitivity** - Probability that a test or procedure **result** is **positive** when the **disease** is **present**; calculated as follows: **number of true-positive results/(number of true-positive results + false-negative results)**

**Specificity** - Probability that a test or procedure **result** is **negative** when **disease** is **not present;** calculated as follows: **number of true-negative results/(number of true-negative results + false-positive results)**

**True-positive rate** - Sensitivity (percentage)

**False-positive rate** - Probability that a test or procedure result is positive when the disease is not present; calculated as follows: **100% minus the specificity (percentage)**

**True-negative rate** - Specificity (percentage)

**False-negative rate** - Probability that a test or procedure result is negative when the disease is present; calculated as follows: **100% minus the sensitivity (percentage)**

**Positive predictive value** - Probability that the disease is present when the test or procedure result is positive; calculated as follows: **number of true-positive results/(number of true-positive results + false-positive results)**

**Negative predictive value** - Probability that the disease is not present when the test or procedure result is negative; calculated as follows: **number of true-negative results/(number of true-negative results + false-negative results)** [361]**.**

**Direct school screening cost:** the cost of the screening program per se; examiners’ salary, transportation, screening equipment.

**Indirect school screening cost:** the cost of false positive results, the follow up visits, the radiographs and the cost of brace treatment and/or surgery. In addition as an indirect cost can be recognized the financial and psychological impact of the screening procedure and the identification of scoliosis on the child and on his or her family [133].
